# Effects of dietary fibers with high water-binding capacity and swelling capacity on gastrointestinal functions, food intake and body weight in male rats

**DOI:** 10.1080/16546628.2017.1308118

**Published:** 2017-04-03

**Authors:** Chengquan Tan, Hongkui Wei, Xichen Zhao, Chuanhui Xu, Jian Peng

**Affiliations:** ^a^ Department of Animal Nutrition and Feed Science, College of Animal Science, South China Agricultural University, Guangzhou, PR China; ^b^ Department of Animal Nutrition and Feed Science, College of Animal Science and Technology, Huazhong Agricultural University, Wuhan, PR China; ^c^ The Cooperative Innovation Center for Sustainable Pig Production, Wuhan, PR China

**Keywords:** Soluble fiber, water-binding capacity, guar gum, food intake, mean retention time

## Abstract

**Objective**: The aim of this study was to investigate the effects of supplementation of dietary soluble fibers with high water-binding capacity (WBC) and swelling capacity (SC) on gastrointestinal tract mass, physicochemical properties of digesta, gastrointestinal mean retention time (MRT), body weight, and food intake in male rats.

**Methods**: Thirty-two male Sprague-Dawley rats were randomized to four equal groups and fed the control diet or diet containing 2% *konjac* flour (KF), pregelatinized waxy maize starch plus guar gum (PWMS+GG), andPWMS plus xanthan gum (PWMS+XG) for three weeks.

**Results**: WBC and SC of diets followed the order of PWMS+GG > KF > PWMS + XG > control. PWMS+GG and KF groups had a lower average daily food intake than the control group, but all the groups showed no difference in final body weightand the weight gain rate. The high WBC and SC of the PWMS+GG and KF groupsled to an increase of WBC and SC in the stomach digesta, and a gain of the cecal digesta weight, due to increased cecal moisture content.

**Conclusion**: The inclusion of the novel fiber, PWMS+GG, in the diet of male rats appears to facilitate the modulation of WBC and SC of stomach digesta and the reduction of food intake.

## Introduction

The chronic nature of obesity and its related diseases suggests the necessity to adopt comprehensive management approaches to achieve and maintain weight loss. One of the approaches is the modulation of food intake by consuming foods with a high satiety value. Previous studies indicated that dietary fiber can reduce food intake and body weight, but the data from human trials are controversial [1,2], probably due to the variability in study designs. In this type of research, animal models are often used due to the advantages of complete control over the diet and collection of gut samples. Studies in rats and mice have shown that diets supplemented with fibers result in lower food intake and body weight, but the efficacy of dietary fibers varies with their amount and composition [3–5]. This could be attributed to the difference in the physicochemical properties of dietary fibers, which are expected to influence digestive physiology in different ways throughout the gastrointestinal tract [6,7].

The addition of soluble fibers increases viscosity and water-binding capacity (WBC) of digesta and induces the formation of gels in the stomach [8,9]. These properties may slow down gastric emptying and concurrently increase stomach distension [10]. Stomach distension, or fullness, is considered as a causal factor in the chain of events leading to satiation [11,12]. In response to the mechanical and physicochemical properties of the ingested foods, a series of neural and humoral signals are generated in the gut to induce satiation [13]. Several studies have investigated the effects of viscous dietary fibers on physiological responses and food intake [10,14], but little information is available on other physicochemical characteristics such as WBC and swelling capacity (SC) of individual or mixed dietary fibers used in different intervention studies.

*Konjac* flour (KF) is derived from the tuber of *Amorphophallus konjac* K. Koch [15], and has been consumed for centuries as rubbery jelly, noodles, or other Asian food products. KF, a water soluble and viscous dietary fiber with a high WBC and SC, mainly contains *konjac* glucomannan [16]. KF has been shown to promote the growth of intestinal mucosa, modify the intestinal microbiota, improve bowel movements, and modulate lipid metabolism [17–19]. Our previous studies have shown that dietary inclusion of KF promoted satiety and reduced food intake, probably due to high WBC and SC in this fiber material [7,20]. However, the tuber is usually grown in Asian countries and the resources for commercial production are limited, suggesting the necessity to develop novel dietary fibers with functional properties similar to those of KF including food intake reduction.

Pregelatinized waxy maize starch (PWMS), a fermentable resistant starch mainly consisting of highly branched amorphous amylopectin, has many specific food attributes [21,22]. Guar gum (GG), a galactomannan extracted from the endosperm of leguminous guar, is widely used as a gelling agent in food industry [23]. Xanthan gum (XG), a biosynthetic edible gum produced by the bacterium *Xanthomonas campestris*, is composed of glucose, mannose and glucuronic acid [24]. Both GG and XG have great viscosity and hydrating properties [25,26]. Low-digestible carbohydrates such as modified starches and gums are often used together in food systems to increase viscosity, retain moisture, improve the overall product quality or stability, and reduce costs or facilitate processing [27–29]. Therefore, we hypothesize that PWMS+GG and PWMS + XG have functional properties similar to KF, and can also reduce food intake of the rats fed those dietary fibers. The objective of this study was to determine the effects of supplementation of dietary soluble fibers with high WBC and SC on gastrointestinal tract mass, physicochemical properties of digesta, mean retention time (MRT), food intake and body weight gain in male rats.

## Materials and methods

### Animals

We only used male rats in this study to focus on the effect of dietary fiber on food intake and its association with physicochemical properties of digesta and gastrointestinal mean retention time. The 32 male Sprague-Dawley rats (average initial weight, 377.10 ± 3.43 g, 10 weeks of age) used in the present study were purchased from Hunan SJA Laboratory Animal Co., Ltd. (license: SCXK (Xiang) 2011–0003). Rats were housed individually in stainless steel wire-bottom cages in a temperature (21 ± 2°C) and humidity (55 ± 10%) controlled facility with 12 h light and dark cycles. Prior to the experiment, rats were fed on the control diet for 7 days and given free access to water.

### Experimental design and treatments

Rats were divided randomly to four dietary treatment groups (eight rats/treatment) and given free access to pelleted diets. Four dietary treatments were utilized in this study: a control diet containing wheat bran as a fiber source (Control); a positive control consisting of the control diet with 2% KF (QINGJIANG KONJAC PRODUCTS CO., LTD., China) to replace 2% wheat bran (KF); a treatment making up the control diet with 2% PWMS (HANGZHOU PULUOXIANG STARCH CORP., LTD., China) plus GG (SHANDONG YUNZHOU SCIENCE AND TECHNOLOGY CORP., LTD., China) to replace 2% wheat bran (PWMS + GG; 85.7% PWMS and 14.3% GG); and a treatment comprising the control diet with 2% PWMS plus XG (SHANDONG YUNZHOU SCIENCE AND TECHNOLOGY CORP., LTD., China) to replace 2% wheat bran (PWMS + XG; 95% PWMS and 5% XG). Chromic oxide was added to the diet at 4 g/kg as an indigestible marker. The ingredients and chemical compositions of the diets are listed in Table 1. The study lasted 21 days. Food intake was recorded daily and body weights were measured at the beginning and the end of the experiment.

**Table 1.** Ingredient and composition of experimental diets.

Ingredient (% w/w)	Control^a^	KF^a^	PWMS +	PWMS + XG^a^
Corn	52.20	52.20	52.20	52.20
Soybean meal	15.90	15.90	15.90	15.90
Fish meal	10.00	10.00	10.00	10.00
Wheat bran	12.00	10.00	10.00	10.00
Sucrose	5.00	5.00	5.00	5.00
Fiber source		2.00	2.00	2.00
Chromic oxide	0.40	0.40	0.40	0.40
AIN-93 Mineral mix^b^	3.50	3.50	3.50	3.50
AIN-93 Vitamin mix^c^	1.00	1.00	1.00	1.00
Composition				
Crude protein (%)	20.04	19.94	19.78	19.90
Energy (kcal/g)	3.81	3.83	3.76	3.74
Fat (%)	4.26	4.26	4.26	4.26
Carbohydrate (%)	58.79	60.29	60.65	60.67
Insoluble fiber (%)	10.46	10.58	10.61	11.10
Soluble fiber (%)	1.91	2.89	3.09	2.57
Viscosity (mPa/s)	1.53	1.66	1.60	1.57
SCd mL/g)	1.81	2.63	3.03	2.18
WBC^e^(g/g)	2.05	2.58	2.87	2.26

^a^Diets were control or supplemented with 2% fiber of *konjac* flour (KF), pregelatinized waxy maize starch plus guar gum (PWMS + GG), or pregelatinized waxy maize starch plus xanthan gum (PWMS + XG).

^b^AIN-93 Mineral mix according to [30], per kg mix: Calcium carbonate, 357.00 g; Potassium phosphate, 196.00 g; Potassium citrate, 70.78 g; Sodium chloride, 74.00 g; Potassium sulfate, 46.60 g; Magnesium oxide, 24.00 g; Ferric citrate, 6.06 g; Zinc carbonate, 1.65 g; Sodium meta-silicate, 1.45 g; Manganous carbonate, 0.63 g; Cupric carbonate, 0.30 g; Chromium potassium sulfate, 0.28 g; Boric acid, 0.08 g; Sodium fluoride, 0.06 g; Nickel carbonate, 0.03 g; Lithium chloride, 0.02; Sodium selenate, 0.01 g; Potassium iodate, 0.01 g; Ammonium paramolybdate, 0.008 g; Ammonium vanadate, 0.007 g; Powdered sucrose, 221.03 g.

^c^AIN-93 Vitamin mix according to [30], g/kg mix: Nicotinic acid, 3.00; Ca pantothenate, 1.60; Pyridoxine, 0.70; Thiamin, 0.60; Folic acid, 0.20; Biotin, 0.02; Vitamin B_12_, 2.50; Vitamin E (500IU/g), Vitamin A (500,000 IU/g), 0.80; Vitamin D_3_ (400,000 IU/g), 0.25; Vitamin K, 0.08; Powdered sucrose, 974.65.

^d^SC: swelling capacity

^e^WBC: water binding capacity.

### Sample collection

After three weeks dietary treatment, rats were fasted overnight and then given food *ad libitum* the next morning and their food intake was recorded. Then, rats were placed in an ethylene oxide chamber and the abdomen was opened by a ventral midline incision. The digestive tract from the stomach through the large intestine was removed. Stomach, small intestine, cecum and colon with contents were weighed to determine the total weight. Next, digesta was separately collected from the stomach and small intestine, and placed in an ice box for viscosity analysis. After collection of the samples, the tissues were cleaned with water, blotted dry, and weighed to determine empty digestive tract weights. Total digestive tract contents were calculated as total tissue weight with contents minus empty tissue weight. All experimental protocols were approved by the animal care and use committee of Huazhong Agriculture University and were in accordance with the National Research Council’s Guide for the Care and Use of Laboratory Animals. All the rats were sacrificed under the supervision of a local animal care committee.

### Physicochemical analysis of digesta and diet

Viscosity in extracts of diets and digesta was measured as previously described [31]. Briefly, 2 g of digesta was centrifuged (10,000 × *g* for 20 min at 4^°^C) to separate solid (sediment) from liquid (supernatant) digesta. The diets (2 g) were dissolved and extracted for 1 h at 40°C in 8 ml of 0.9% sodium chloride with 0.02% sodium azide (NaCl + NaN_3_) and centrifuged at 10,000 × *g* and 4^°^C for 20 min. After centrifugation, the supernatant fraction of the digesta was removed by suction, and the viscosity of the supernatant was measured in a Brookfield DV-11 cone/plate viscometer (Brookfield Engineering Laboratories Inc., Middleboro, MA 02346, USA) at 38^°^C in the shear rate range of 2.25 to 450 s^−1^. The remaining supernatant was frozen and stored for subsequent analyses.

SC was measured as previously described [32]. Briefly, 300 mg of sample was dissolved in 10 ml of NaCl + NaN_3_ and placed in a shaking water bath at 150 movements/min and 39^°^C for 20 h. The SC [ml/g of dry matter (DM)] was measured 1 h after turning off the water bath.

The WBC (g of water/g of DM) of the sediment fraction of digesta was calculated according to the following equation [32]:

(1) 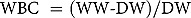

Where WW is the wet weight, and DW is the dry weight of the material.

### Mean retention time analysis

The digesta from the digestive tract including stomach and small intestine was collected and freeze-dried. After drying, samples were ground with a coffee grinder to pass through a 1-mm screen, weighed to obtain dry digestive tract content, and used for analysis of Cr_2_O_3_ concentration in digesta and feed. MRT was calculated using the following equation:

(2) 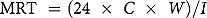

Where MRT is the mean retention time (h); *C* is the Cr_2_O_3_ concentration in the digesta (mg/g); *W* is the weight of the dry digestive tract content (g); and *I* is the intake over 24 h (mg food intake × Cr_2_O_3_ concentration in the feed) and 24 equals h/d [33].

### Chemical analysis

The dry matter of diet or cecal digesta samples was obtained by drying them (mostly 20 h) to a constant weight at 103^°^C, and crude protein (N × 6.25) was determined by the Kjeldahl method (reference no. 978.02; AOAC, 1990). Gross energy was determined by bomb calorimetry using a LECO Ac 300 automated calorimeter system (LECO, St. Joseph, MI). Soluble dietary fiber and insoluble fiber were determined by AOAC Method 991.43 (1990). The chromium in the diet and digesta samples was determined using an Instrumentation Laboratory atomic absorption spectrophotometer as described by Costigan and Ellis [34]. All procedures were performed in duplicate.

### Statistical analyses

Data were analyzed as a completely randomized design using the Mixed Models procedure of SAS (SAS Institute, Inc., Cary, NC, USA). The model contained the fixed effect of diet and the random effect of rat. Differences among treatments were determined using a Fisher-protected least significant difference test with a Tukey adjustment to control for experiment-wise error. Data were presented as means ± SEM, and significant differences were accepted at a probability of *P *≤ 0.05.

## Results

### Physicochemical properties of fiber materials and diets, body weight, and food intake

The physicochemical properties of diets are shown in Table 1. The highest WBC and SC were found in the PWMS + GG diet, followed by KF diet, and PWMS + XG diet, with the lowest WBC and SC in the control diet, indicating the WBC and SC were higher in fiber materials than in the wheat bran. As shown in Table 2, the PWMS + GG, KF and PWMS + XG showed significantly higher WBC (*P *< 0.01) and SC (*P *< 0.01) than the wheat bran (Table 2). Although the viscosity for wheat bran was lower than that of the other fiber materials (*P *< 0.01), the viscosity was not significantly different among the four dietary treatments.

**Table 2.** Physicochemical properties of fiber materials^a^.

Item	WB^b^	KF^b^	PWMS + GG^b^	PWMS + XG^b^	*P*-value
Viscosity(mPa/s)	1.11 ± 0.10^e^	13.63 ± 0.94^d^	19.80 ± 0.67^c^	14.93 ± 2.93^d^	<0.0001
SC (mL/g)	2.08 ± 0.01^e^	23.84 ± 0.45^d^	27.41 ± 0.92^c^	23.22 ± 0.58^d^	<0.0001
WBC (g/g)	3.92 ± 0.11^e^	23.42 ± 0.15^d^	29.46 ± 1.97^c^	24.03 ± 0.91^d^	<0.001

^a^Fiber materials were determined on a dry basis. Values are mean ±SEM, n = 3 per group.

^b^WB: wheat bran, KF: *konjac* flour, PWMS: pregelatinized waxy maize starch, GG: guar gum, XG: xanthan gum, SC: swelling capacity; WBC: water binding capacity.

^c,d,e^means values with different superscript letters in the same row are significantly different (*P* < 0.05).

Daily food intake, body weight, and weight gain rates are presented in Table 3. The initial body weights of the rats were similar among the four groups and, after 21 days on the experimental diets, the final body weights and weight gain rates did not differ significantly. Compared with the control group, the daily food intake was obviously lower in the PWMS + GG and KF groups (*P *= 0.05), but there was no significant difference between the control and PWMS + XG groups.

**Table 3.** Daily food intake, weight gain rate, and final body weight of rats fed different dietary fibers^a^.

Item	Control^b^	KF^b^	PWMS + GG^b^	PWMS + XG^b^	*P*-value
Daily food intake, g/day	37.81 ± 0.61^c^	34.71 ± 0.69^d^	35.16 ± 0.98^d^	36.29 ± 0.97^cd^	0.05
Initial body weight, g	376.31 ± 7.01	377.88 ± 5.90	374.25 ± 8.89	379.81 ± 6.53	0.96
Final body weight, g	503.00 ± 9.74	492.75 ± 11.14	487.00 ± 11.69	504.00 ± 12.60	0.43
Rate of weight gain, g/day	6.03 ± 0.24	5.47 ± 0.42	5.37 ± 0.25	5.91 ± 0.40	0.63

^a^Values are mean ±SEM, n = 8 per group;

^b^ Diets were control or supplemented with 2% fiber of *konjac* flour (KF), pregelatinized waxy maize starch plus guar gum (PWMS + GG), or pregelatinized waxy maize starch plus xanthan gum (PWMS + XG);

^c,d^ means values with different superscript letters in the same row are significantly different (*P *< 0.05).

### Digestive tract mass, digesta weight, and cecal moisture content

**Figure 1.**
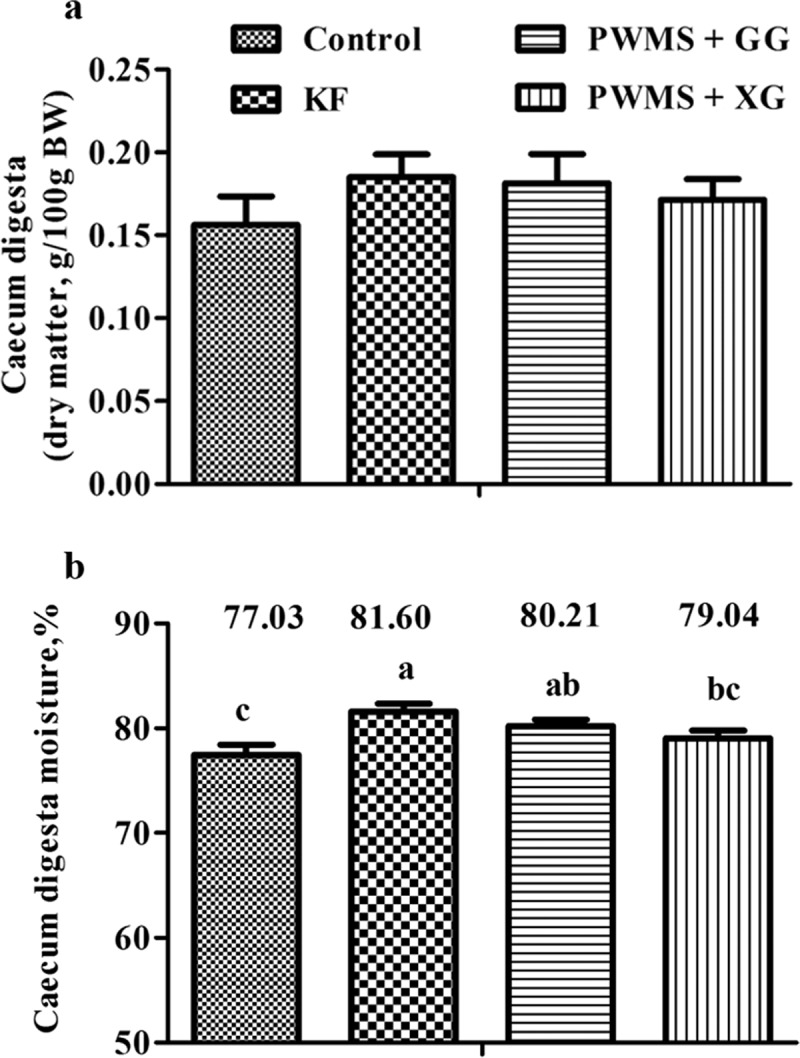
Cecal digesta weight (dry matter, A) and moisture content (%, B) of rats fed different dietary fibers. Values are mean ±SEM, n = 8 per group. Diets were control or supplemented with 2% fiber of konjac flour (KF), pregelatinized waxy maize starch plus guar gum (PWMS + GG), or pregelatinized waxy maize starch plus xanthan gum (PWMS + XG). a,b means values with different letters in the bar are significantly different (*P *< 0.05).

The four diet groups showed no significant differences in tissue and fresh digesta weights of stomach, small, and colon (Table 4). The empty cecum weight was also similar among the four diet groups. However, the fresh digesta weight in cecum was increased (*P *< 0.05) as a result of consumption of PWMS + GG and KF. This effect was not noted in the PWMS + XG group when compared to the control group. Although dry matter of cecal digesta showed no obvious differences among the four diet groups (Figure 1), the cecal moisture content was significantly higher (*P *< 0.05) in the PWMS + GG and KF groups than in the control group.

**Table 4.** Gastrointestinal tissue and fresh digesta masses of rats fed different dietary fibers^a^.

Item	Control^b^	KF^b^	PWMS + GG^b^	PWMS + XG^b^	*P*-value
Stomach (g/100 g BW)					
Tissue	0.45 ± 0.03	0.49 ± 0.03	0.48 ± 0.02	0.47 ± 0.03	0.85
Digesta, fresh	2.27 ± 0.23	2.18 ± 0.39	2.25 ± 0.36	2.10 ± 0.29	0.98
Small intestine (g/100 g BW)					
Tissue	2.07 ± 0.10	2.18 ± 0.10	2.07 ± 0.09	2.15 ± 0.13	0.85
Digesta, fresh	0.93 ± 0.05	1.11 ± 0.10	1.05 ± 0.09	1.01 ± 0.08	0.48
Caecum (g/100 g BW)					
Tissue	0.39 ± 0.03	0.35 ± 0.01	0.37 ± 0.02	0.36 ± 0.02	0.62
Digesta, fresh	0.69 ± 0.06^d^	1.01 ± 0.06^c^	0.91 ± 0.08^c^	0.82 ± 0.06^cd^	0.01
Colon (g/100 g BW)					
Tissue	0.28 ± 0.02	0.31 ± 0.03	0.31 ± 0.03	0.31 ± 0.03	0.84
Digesta, fresh	0.35 ± 0.04	0.47 ± 0.05	0.48 ± 0.08	0.41 ± 0.19	0.42

^a^Values are mean ±SEM, n = 8 per group;

^b^ Diets were control or supplemented with 2% fiber of *konjac* flour (KF), pregelatinized waxy maize starch plus guar gum (PWMS + GG), or pregelatinized waxy maize starch plus xanthan gum (PWMS + XG);

^c,d^ means values with different superscript letters in the same row are significantly different (*P *< 0.05).

### Viscosity, WBC, SC, and MRT of stomach and small intestine digesta

**Figure 2.**
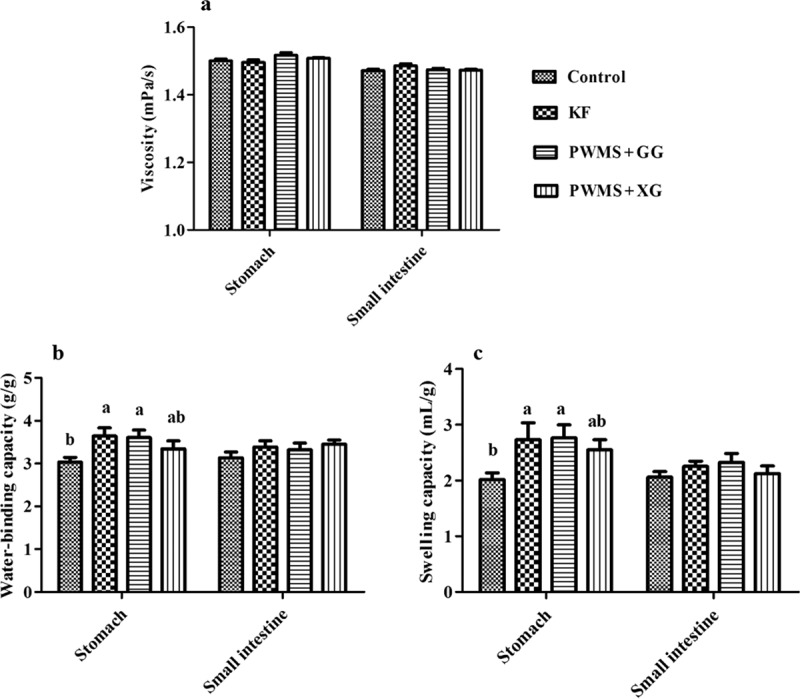
The viscosity (A), water-binding capacity (B) and swelling capacity (C) of digesta in the stomach and small intestine of rats fed different dietary fibers. Values are mean ±SEM, n = 8 per group. Diets were control or supplemented with 2% fiber of konjac flour (KF), pregelatinized waxy maize starch plus guar gum (PWMS + GG), or pregelatinized waxy maize starch plus xanthan gum (PWMS + XG). a,b means values with different letters in the bar are significantly different (*P *< 0.05).

**Figure 3.**
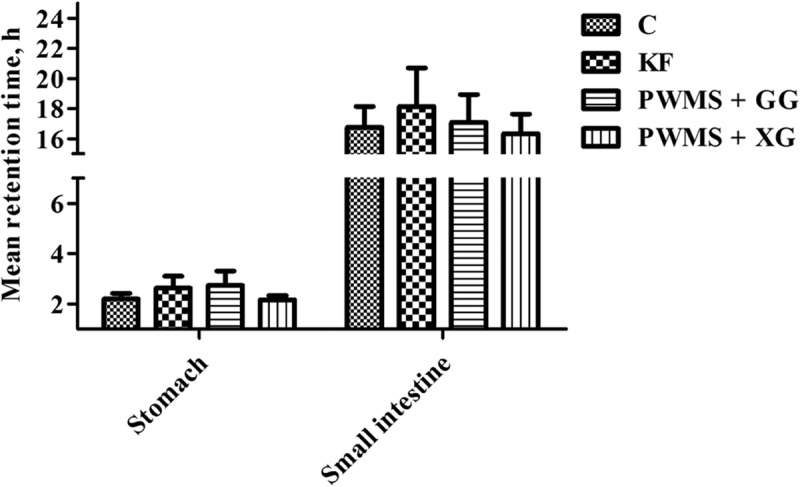
Mean retention time in the stomach and small intestine of rats fed different dietary fibers. Values are mean±SEM, n = 8 per group. Diets were control or supplemented with 2% fiber of konjac flour (KF), pregelatinized waxy maize starch plus guar gum (PWMS + GG), or pregelatinized waxy maize starch plus xanthan gum (PWMS + XG).

The four diet groups also showed no significant differences in stomach and small intestine digesta viscosity (Figure 2). The WBC and SC in small intestine digesta were also similar among all the four groups. However, compared with the control group, the PWMS + GG and KF groups had obviously higher WBC and SC (*P *< 0.05) in stomach digesta, but not the PWMS +XG group. Compared with the control group, the MRT in the stomach tended to prolong in the KF and PWMS + GG groups, but the difference was not statistically significant (*P *= 0.09) (Figure 3).

## Discussion

This study indicated that dietary supplementation with PWMS + GG and KF increased the WBC and SC in stomach digesta as well as the cecal moisture content, and reduced daily food intake in male rats.

Our results showed that fiber materials of PWMS + GG, PWMS + XG and KF had higher WBC and SC than wheat bran. PWMS is a modified starch, and the GG or XG is a gum. Addition of a small amount of gums results in a large WBC enhancement of starch pastes and a reduction of syneresis in starch gels, and the magnitude of such changes in paste rheology and texture depends on the type of starch and gum used [35,36]. The current results (the PWMS + GG group with highest WBC and SC) can be attributed to the retardation of gelation kinetics of waxy maize starch plus the GG, rather than the combination with other gums [37].

In the present study, the WBC and SC of diets followed the order of PWMS + GG > KF > PWMS + XG > control. The physicochemical properties of the diet are linked to the type of polymers that make up the cell wall and are the main factors influencing WBC and SC. Consistent with the study by Serena et al. [38], dietary soluble fiber content has a considerable impact on the physicochemical properties of the diets, and both WBC and SC were increased in the and PWMS + GG diets with the high content of soluble fiber.

The physicochemical properties of dietary fiber may affect postprandial satiety in animals by altering the digestive physiology [39,40]. Both WBC and SC were increased in KF and PWMS + GG diets with the high content of soluble fiber, which may also lead to the increase of WBC and SC in stomach digesta. The increased WBC and SC of digesta could slow down gastric emptying and concurrently increase stomach distension, which may trigger afferent vagal signals of fullness [41]. The present data showed that dietary PWMS + GG or KF tended to prolong the stomach MRT of rats. Insoluble fiber has a direct physical effect in the hindgut because of its bulking effect, and will stimulate propulsive colonic motility and shorten the MRT [42]. In contrast, soluble fiber may lengthen the MRT of digesta owing to its effect on viscosity and WBC [42]. In the current study, dietary supplementation of PWMS + GG or KF reduced daily food intake of rats, probably due to the similar reasons in a previous study that feeding diets rich in soluble fiber reduced the physical activity of sows and the satiating property could be attributed to increased WBC and SC in the stomach, which in turn delayed the gastric emptying rate of the liquid phase of gastric content [43].

Additionally, dietary supplementation of PWMS + GG or KF does not affect the digestive tract of rats or their digesta weight except cecal digesta, probably due to the increase of cecal moisture content. The higher cecal moisture content would be accompanied by an increase of bacterial mass from fermentation [44]. Our previous study showed that dietary fibers like the KF or PWMS + GG group can be fermented in the distal small intestine, leading to the formation of short-chain fatty acids, which may also enhance satiety [20]. Furthermore, dietary fiber may play a significant role in the management of constipation, and improve stool bulking by increasing the moisture content in cecum [45].

In this study, although wheat bran was lower than the other fiber materials in viscosity, the four dietary treatments showed similar viscosity, and thus no differences in the viscosity of digesta. This was in line with our previous study in that the inclusion of 2.1% KF in the gestation diet of sows did not increase the diet viscosity [46].

At 10 weeks of age, the male rats were young adults. They may have ceased the juvenile rapid growth phase, but continue to gain body mass at a slower rate [47]. Therefore, the rats in all the dietary treatments showed a similar weight gain rate, possibly resulting from the short-term effects of different diets.

PWMS + XG did not perform as well as PWMS + GG or KF, probably due to the lower WBC and SC in the former than in the latter.

The overall results from this study indicate that dietary supplementation of KF might reduce daily food intake in rats by increasing the WBC and SC of stomach digesta. Additionally, PWMS + GG is similar to KF in the effect on food intake, but not the PWMS + XG diet. The novel fiber, PWMS + GG appears to promote the modulation of WBC and SC of stomach digesta and the reduction of food intake when supplemented in the diet of the male rats.
